# Challenges in prenatal care of ectopic cordis: Case series and literature review

**DOI:** 10.1016/j.radcr.2025.06.080

**Published:** 2025-08-05

**Authors:** Abarham Martadiansyah, Peby Maulina Lestari, Nuswil Bernolian, Putri Mirani, Muhammad Al Farisi Sutrisno, Hana Andrina, Ingrid Helena Cristiani Siregar, Efi Kardiana, Bella Stevanny

**Affiliations:** a Division of Fetomaternal Medicine, Department of Obstetrics and Gynecology, Faculty of Medicine, Sriwijaya University, Moh. Hoesin General Hospital, Palembang, South Sumatra, Indonesia; b Department of Obstetrics and Gynecology, Faculty of Medicine, Sriwijaya University, Moh. Hoesin General Hospital, Palembang, South Sumatra, Indonesia

**Keywords:** Congenital anomaly, Ectopic cordis, Neonatal outcome, Pentalogy of Cantrell, Prenatal diagnosis, Ultrasound

## Abstract

Ectopic cordis (EC) is an exceptionally rare anomaly in which the fetal heart lies outside the thoracic cavity, making prenatal recognition essential. We describe 2 prenatally diagnosed cases managed at our tertiary center. The first case involved a 17-year-old primigravida carrying a 36-week female fetus with complete EC and an intact diaphragm. The second case concerned a 27-year-old primigravida whose 35-week female fetus exhibited partial EC, a ventricular septal defect, lower sternal cleft, and an omphalocele consistent with Pentalogy of Cantrell. After cesarean delivery and temporary heart coverage, the first neonate died on day 12, whereas the second neonate survived and was discharged on day 10 following conservative stabilization. These contrasting outcomes show that prognosis hinges on the degree of cardiac displacement and accompanying defects, underscoring the importance of early prenatal detection and coordinated multidisciplinary care to optimize outcomes.

## Introduction

Ectopic cordis (EC) is a rare congenital anomaly that affects the heart. It is characterized by a defect in the chest and abdominal wall, resulting in the heart being positioned outside the thoracic cavity. This condition also involves abnormalities in the pericardium, diaphragm, sternum, and often includes cardiac malformations. The displacement of the heart from its normal location within the thoracic cavity is indicative of Ectopic cordis. The historical origins of Ectopic cordis date back to 5000 years ago, and the term "ectopic cordis" was officially coined by Haller et al. in 1706. Ectopic cordis usually occurs sporadically, but there have been documented cases associated with chromosomal anomalies such as trisomy 18, Turner syndrome, 46,XX, and 17q+. As outlined by Khoury, the incidence of ectopic cordis (EC) is estimated to be as low as 8 cases per 1,000,000 live births, with 65% of these cases being of the thoracic type [1,2].

Pentalogy of Cantrell is a rare congenital syndrome involving defects in the lower sternum, anterior diaphragm, abdominal wall, ectopic cordis, and congenitaly heart disease. First described by Cantrell in 1958, the etiology is unknown but often associated with aneuploidy and failure of lateral mesodermal folds during early embryogenic development. The prevalence is estimated 1 in 65,000 to 1 in 200,000 births, with less than 100 cases reported globally. Prenatal diagnosis is typically obtained using Color Doppler ultrasound in the second trimester. Management involves multidisciplinary disciplined and surgical repair immediately after birth. Prognosis depends on the severity of ectopic cordis and related anomalies, with a survival rate of approximately 37%. Complications may include cardiac trauma and the surgical repair is challenging according to the size of the defect [3,4].

Clinical presentations described in multiple cases have been centered around intrinsic cardiac abnormalities and sepsis. Infants commonly exhibit symptoms of breathlessness due to disruptions in the heart's pumping mechanism linked to inherent anomalies. The presence of fever can be attributed to the lack of skin and pericardium, rendering the individual vulnerable to bacterial infections and potentially leading to sepsis. The challenge in managing sepsis arises when the atmospheric contact is not restored, making treatment more complex [[5], [6]].

In this report, we present 2 cases of prenatally diagnosed ectopic cordis with differing phenotypic severity and outcomes: 1 case of complete EC without associated defects and 1 case of partial EC consistent with POC. Through these cases, we aim to highlight the challenges in prenatal imaging, perinatal decision-making, and postnatal outcomes associated with this rare and life-threatening condition.

## Case presentation

This study is a retrospective case series performed at a single center in Palembang involving non-consecutive cases. We retrospectively examined 2 cases of ectopic cordis: 1 case with complete displacement of the heart and another with partial displacement, accompanied by abnormalities in the lower sternum, anterior diaphragm, and abdominal wall, consistent with Pentalogy of Cantrell.

### Case number 1

#### Prenatal evaluation

A 17-year-old primigravida at 35 weeks’ gestation was referred from a rural hospital with a presumptive diagnosis of fetal ectopic cordis. Her antenatal care began at 16 weeks under the supervision of a midwife, with monthly visits. No significant abnormalities were noted during those early evaluations. She had no history of chronic illnesses (such as diabetes or hypertension), prior surgeries, or exposure to teratogenic substances. The patient denied tobacco, alcohol, or illicit drug use. There was no family history of congenital anomalies, genetic disorders, or ectopic cordis. She had not undergone any genetic screening or early anomaly scans. At 24 weeks’ gestation, she visited an obstetrician for the first time, and a fetal anomaly was suspected. Ultrasound revealed an anterior thoracic mass consistent with an extracorporeal heart. Referral to a tertiary center was advised, but the family declined. A repeat scan at 33 weeks by another obstetrician confirmed persistent ectopic cordis. At this point, the patient agreed to be referred to our tertiary institution for further evaluation.

**Fig. 1:**
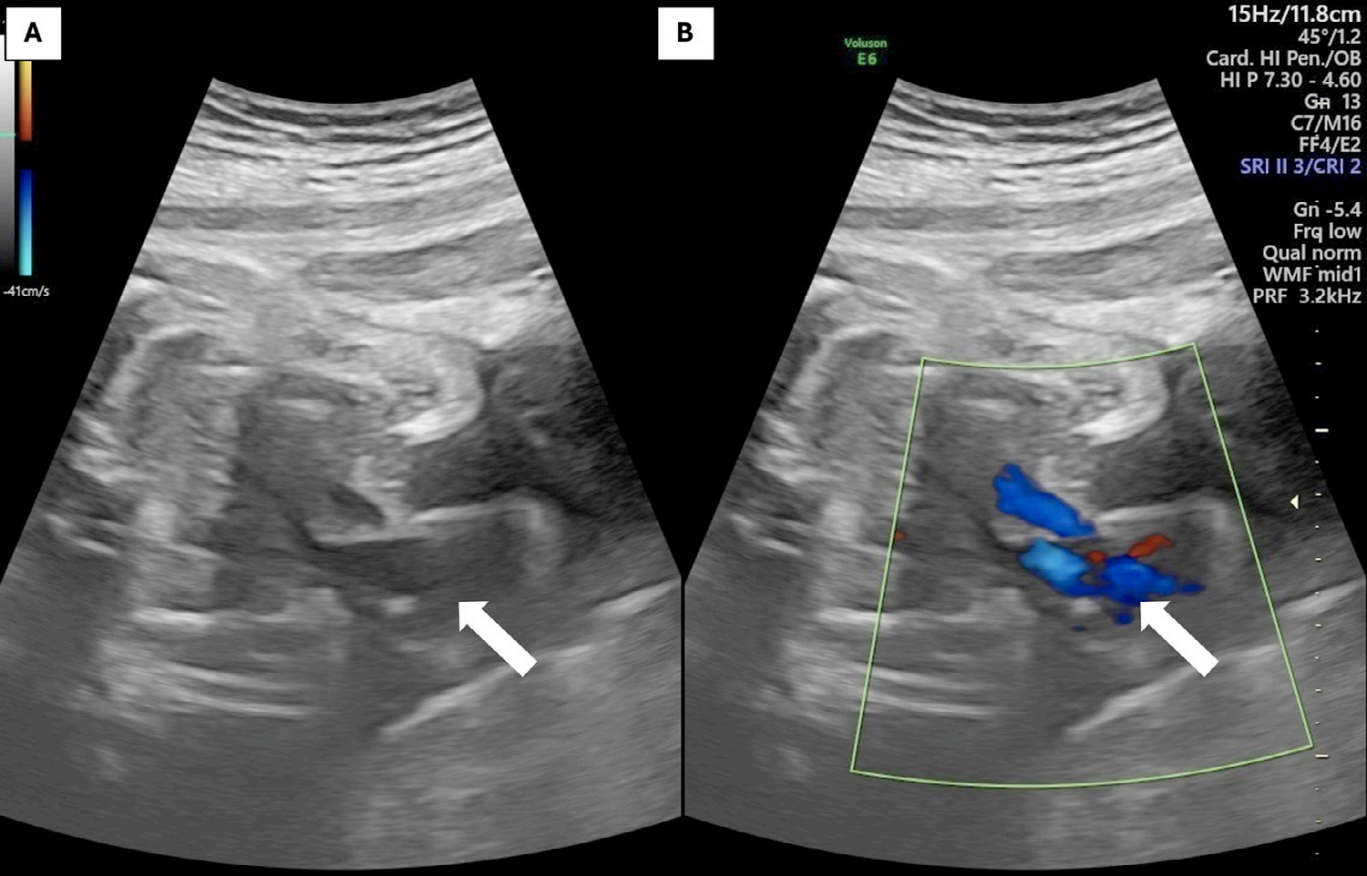
An ultrasound anomaly scan of a 36-week fetus with ectopic cordis reveals the heart entirely located outside the chest cavity (white arrow).

**Fig. 2:**
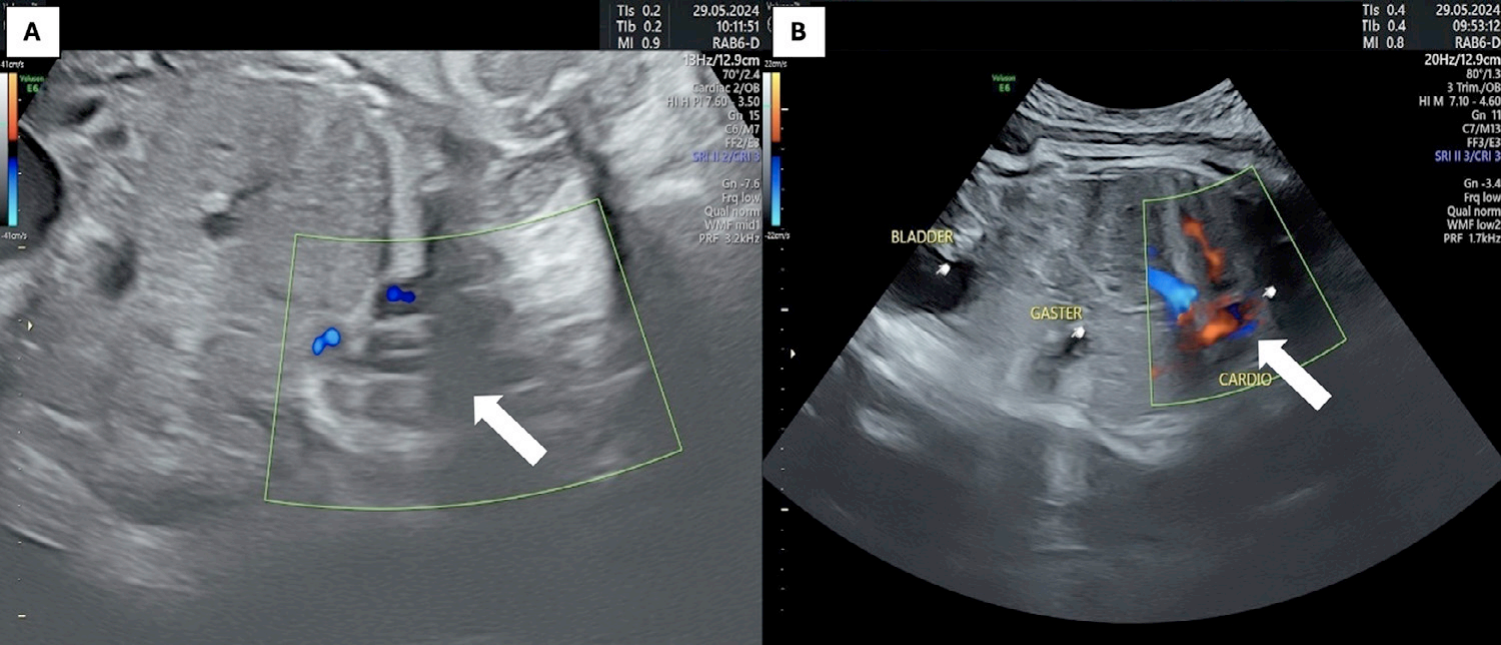
Ultrasound showing that atriums and ventricles lie outside the chest cavity the heart is located completely outside the chest cavity (white arrow), with a normal 4-chamber appearance and a regular heartbeat. In the abdomen, the stomach was normal, the diaphragm was intact, the kidneys and bladder were normal during sonography and normal umbilical cord insertion was noted.

Upon presentation at our center, physical examination of the mother was unremarkable. Vital signs were stable. Antenatal ultrasound at 36 weeks revealed a normal singleton fetus in cephalic presentation, with a structurally normal brain, spine, extremities, kidneys, and bladder. Cardiac assessment showed a structurally normal 4-chamber heart completely external to the thoracic cavity, with no pericardial covering or thoracic wall protection (Fig. 1). Fig. 2 illustrates normal fetal biometry with intact diaphragm and umbilical insertion. No intracardiac anomalies were identified. The diaphragm appeared intact. Estimated fetal weight was appropriate for gestational age. Routine laboratory tests for the mother showed Hemoglobin: 11.8 g/dL (normal: 11-13 g/dL); Leukocytes: 7,200/mm³ (normal: 4,000-10,000/mm³); Platelets: 250,000/mm³ (normal: 150,000-450,000/mm³); Blood glucose: 92 mg/dL (normal: 70-110 mg/dL); with no proteinuria or abnormal liver/renal function noted.

#### Delivery and postnatal course

**Fig. 3:**
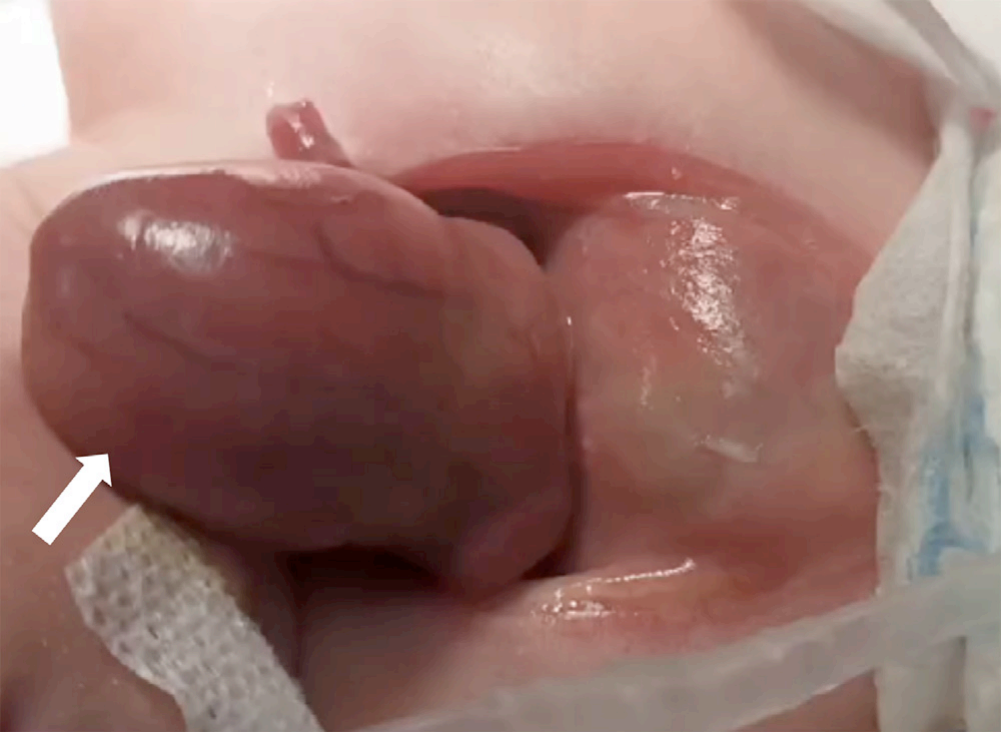
External heart located entirely outside the chest cavity without pericardium.

A multidisciplinary team decided to proceed with delivery by lower segment cesarean section (LSCS) at 36 weeks. A female neonate was born alive, weighing 1,955 g, with Apgar scores of 8 and 9 at the 1st and 5th minutes, respectively. Postnatal examination confirmed complete ectopia of the heart, with no overlying thoracic wall or pericardium, as seen in Fig. 3. No omphalocele or other gross structural anomalies were noted. No chromosomal studies could be performed due to financial and logistical limitations.

Initial stabilization included gentle coverage of the exposed heart with warm saline-soaked sterile gauze and plastic wrap. The neonate was transferred to the NICU, where further resuscitative care was given. Surgical management included placement of a Bogota bag and bovine pericardium patch for temporary protection of the heart.

#### Outcome

Despite supportive care, the newborn developed progressive respiratory distress and sepsis. Blood cultures were positive for Klebsiella pneumoniae. Antibiotic therapy (meropenem and amikacin) and supportive management were initiated, but the neonate deteriorated rapidly and passed away on postnatal day 12 due to multiorgan failure.

In addition to the static ultrasound images provided, a supplementary video (Supplementary Video 1) demonstrates dynamic fetal echocardiographic findings of the ectopic heart with free extracorporeal motion. This visual highlights the severity of cardiac displacement and supports prenatal imaging assessment for EC.

### Case number 2

#### Prenatal evaluation

A 27-year-old primigravida at 35 weeks’ gestation was referred with suspected partial ectopic cordis. Antenatal care had been initiated at 8 weeks by an obstetrician, with regular visits every 1-2 months. There was no significant past medical or surgical history. She denied use of alcohol, tobacco, or other substances. No history of consanguinity or familial congenital disorders was reported.

**Fig. 4:**
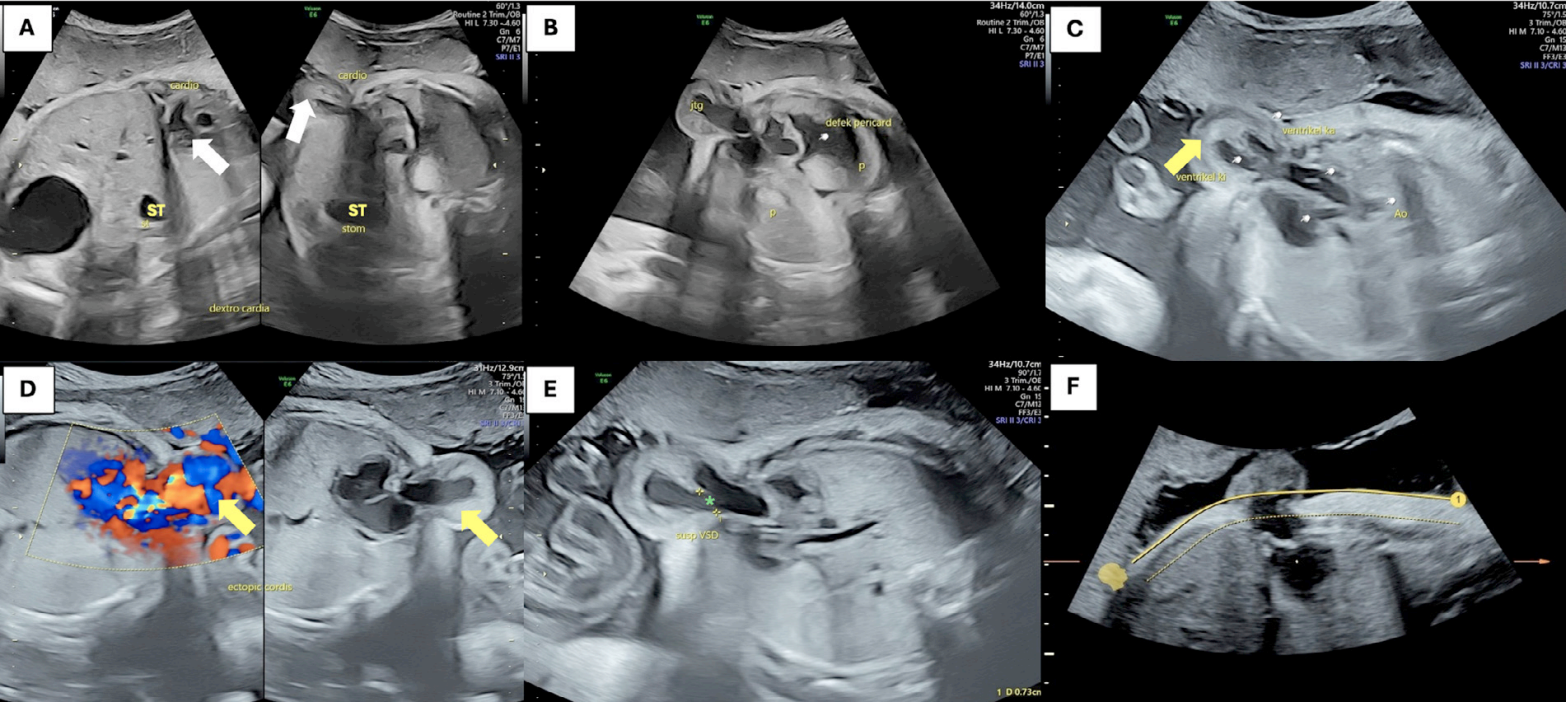
(A) Ultrasound shown the position of the heart (white arrow) is to the right of the stomach (ST) leading to dextrocardiac, (B) absence of the pericardium refer to pericardial defect, (C & D) part of the ventricle (yellow arrow) is located outside thoracic cavity refer to partial ectopic cordis, (E) ventricular septum defect (green asterisk) approximately 7 mm, (F) chest wall deformity refer to lower sternal cleft.

**Fig. 5:**
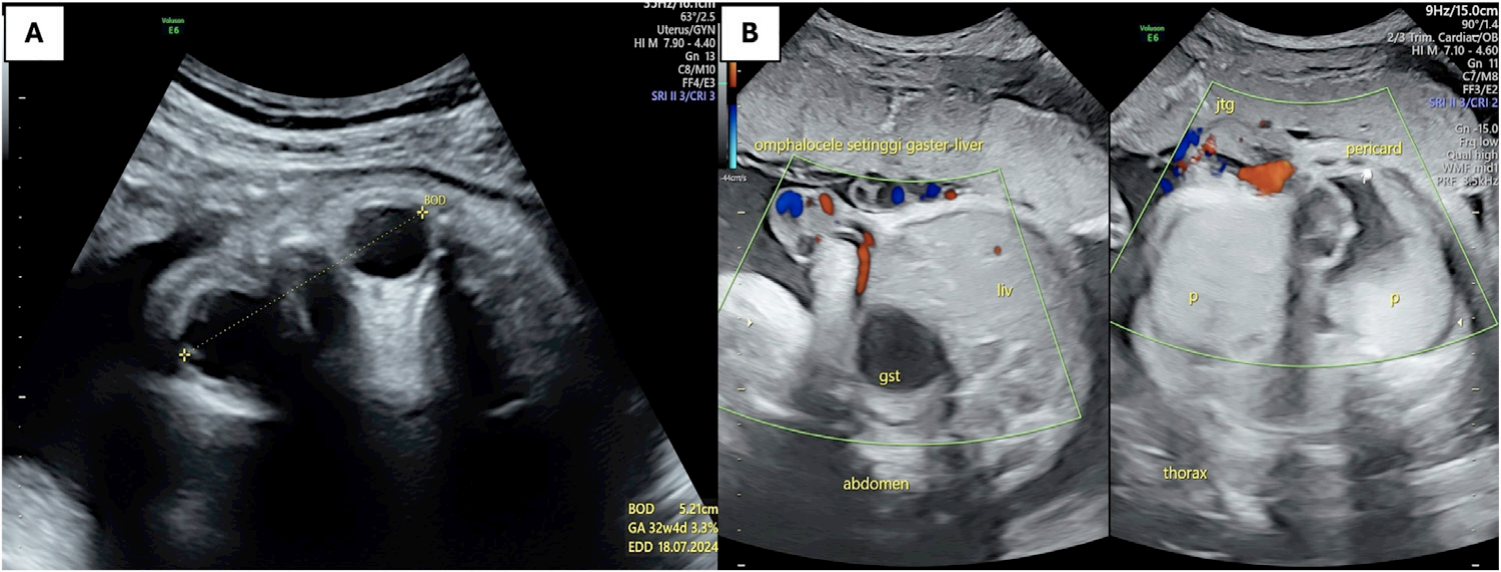
(A) Ultrasound show binocular distance 5.21 cm result to hypotelorism and (B) supraumbilical omphalocele at the level of the stomach-liver.

At 32 weeks, ultrasound performed at a district hospital revealed cardiac displacement and prompted referral. Upon evaluation at our center, a comprehensive ultrasound revealed multiple midline anomalies: dextrocardia with rightward displacement of the heart (Fig. 4A), pericardial defect (Fig. 4B), and partial extracorporeal ventricular position consistent with partial ectopic cordis (Fig. 4C-D). A ventricular septal defect measuring 7 mm was identified (Fig. 4E), along with a lower sternal cleft (Fig. 4F). Hypotelorism was noted on biometric assessment (Fig. 5A), and a supraumbilical omphalocele was seen protruding at the level of the stomach and liver (Fig. 5B). These findings were consistent with Pentalogy of Cantrell (class II). Maternal lab values were within normal limits with Hemoglobin: 12.1 g/dL; Leukocytes: 6,800/mm³; Platelets: 273,000/mm³; Fasting glucose: 89 mg/dL; with no evidence of preeclampsia or infection.

#### Delivery and postnatal course

**Fig. 6:**
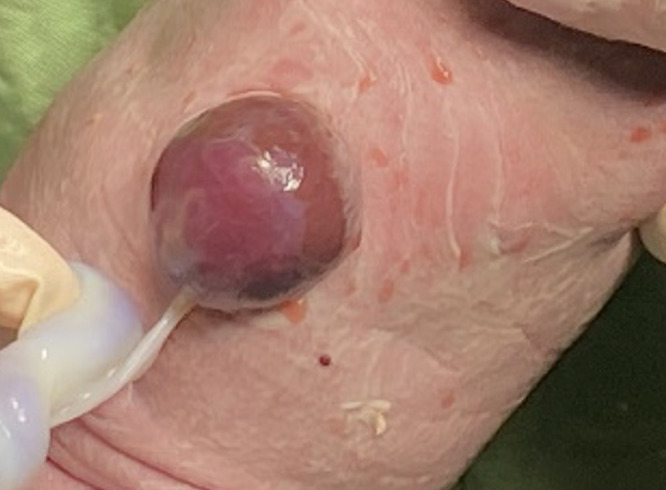
Partial ectopic cordis located outside the chest.

Given the anticipated complexity of delivery, a multidisciplinary team recommended elective LSCS at 38 weeks. A live female infant was delivered with a birth weight of 2,800 g and Apgar scores of 7 and 8 at 1 and 5 minutes. The heart was partially exteriorized without pericardial covering; additionally, a paraumbilical membrane-covered intestinal segment (∼2 cm) was visible, consistent with omphalocele (Fig. 6). There were mild intercostal retractions and low oxygen saturation; however, spontaneous respiration and cardiac function remained stable.

Postnatal management included coverage with saline-moistened sterile dressing and transfer to NICU. A Bogota bag was placed for temporary containment. No surgical repair was performed in the immediate neonatal period. Genetic testing was recommended but declined by the family due to financial and logistical limitations.Echocardiography confirmed VSD, and conservative monitoring was continued.

#### Outcome

The neonate remained hemodynamically stable and gradually improved without surgical repair during the initial admission. She was discharged home on day 10 in good condition, with planned cardiology and surgical follow-up.

## Ethics statement

Written informed consent was obtained from the legal guardians of both patients for publication of the case details and associated images. This case report was prepared in accordance with the CARE guidelines 2013 [7]. Institutional ethics committee approval was not required for publication of de-identified retrospective case reports under local hospital policy.

## Discussion

Ectopic cordis (EC) is a rare congenital anomaly marked by partial or complete displacement of the heart outside the thoracic cavity. It could be occurred as an isolated malformation or associated with defects in the chest, abdominal walls, or both of them. The condition is associated to malformations of the anterior chest wall, with the heart located outside the chest cavity [7]. Based on the heart's position, ectopic cordis is categorized into 5 types: cervical (5%), cervicothoracic and thoracic (65%), thoracoabdominal (20%), and abdominal (10%) [[8], [9]]. As of 2001, 267 cases had been documented, with thoracic (39.2%) and thoracoabdominal (38%) types being most common. Unfortunately, 90% of affected infants do not survive beyond their first year of life. Prenatal diagnosis via ultrasound (USG) is essential for visualizing the heart outside the chest cavity, enabling early detection of ectopic cordis and associated anomalies as early as the second trimester [10,11].

Pentalogy of Cantrell (POC) encompasses 5 congenital anomalies that affect the midline structures of the body, including the heart, pericardium, diaphragm, sternum, and abdominal wall. This condition is classified into 2 categories: complete and partial. Complete POC involves all 5 defects, whereas partial POC includes fewer than 5 anomalies. It is also referred to as thoracoabdominal ectopic cordis, where the heart is encased in a membrane similar to an omphalocele. Ectopic cordis (EC) is commonly linked to POC, and newborns usually have diverse cardiac anomalies, with ventricular septal defects and Tetralogy of Fallot being the predominant ones [9,10]. The complete POC is distinguished by 2 main abnormalities: ectopic cordis and an abdominal wall defect, typically an omphalocele, although gastroschisis may also be present. Furthermore, apart from these, POC encompasses 3 additional abnormalities that impact structures situated between the distal sternum, anterior diaphragm, and diaphragmatic pericardium. The classification of POC includes Class 1, where all 5 defects are present and confirmed; Class 2, where 4 defects are present, including intracardiac and ventral wall abnormalities, suggesting a probable diagnosis; and Class 3, which denotes partial POC with various combinations of existing defects, including sternal abnormalities. The Pentalogy of Cantrell becomes apparent at the time of birth. This disease affects around 1 in 65,000 live newborns. A study conducted in the Baltimore-Washington area on children with congenital heart abnormalities indicated that the prevalence rate in the region is 5.5 cases per 1 million live births. The occurrence of this condition is more frequent in males, with a male-to-female ratio of 1.35-1.15, respectively [11,12].

While the existing literature describes the embryologic origins of EC and associations with POC, few studies provide direct clinical comparisons between complete and partial presentations in modern prenatal and surgical contexts. Our 2 cases illustrate this contrast. The first case—complete EC without intracardiac defects or omphalocele—resulted in early neonatal death despite surgical stabilization. The second case, with partial EC and associated anomalies (VSD, lower sternal cleft, pericardial defect, and omphalocele), was consistent with Toyama Class II POC and had a favorable short-term outcome following conservative management. Notably, the prognosis of EC correlates more strongly with the extent of displacement and associated defects than with gestational age or delivery method. Our second case, despite having multiple anomalies, survived because of partial displacement and intact cardiac function.

Our findings align with the outcomes reported in prior literature. Fazae et al. [13] described a surviving newborn with partial EC and POC Class II features managed successfully with surgical intervention. In contrast, Mraihi et al. [14] reported 2 cases of EC: 1 complete and 1 partial, both of which resulted in early neonatal death despite prenatal detection. Similarly, Limanto et al. [15] reported a case of complete EC without associated anomalies, in which the neonate died on day 3 despite surgical repair. Table 1 provides a summary comparison of previous reports and our cases.

**Table 1:** Comparative summary of reported ectopic cordis cases.

Case	Type of ectopic cordis	Associated anomalies	Toyama class	Delivery	Management	Survival
Present case 1	Complete	None	Not classified	Cesarean	Bogota bag + bovine pericardium	Passed away on Day 12
Present case 2	Partial	VSD, sternal cleft, pericardial defect, omphalocele	Class II	Cesarean	Bogota bag only	Stable and discharged
Fazae et al. [13]	Partial	Omphalocele, VSD, sternal defect	Class II	Cesarean	Surgical repair	Stable and discharged
Mraihi et al. [14], (Case 1)	Complete	Omphalocele, diaphragmatic hernia	Class I	Cesarean	None	Passed away
Mraihi et al. [14], (Case 2)	Partial	EC, omphalocele, diaphragmatic hernia	Class II	Cesarean	None	Passed away
Limanto et al. [15]	Complete	None	Not stated	Cesarean	Surgical repair	Passed away on day 3

This comparison underscores that complete EC, even when isolated, is often fatal, while partial EC with POC Class II anomalies can have better outcomes when cardiac function is preserved and perinatal care is well coordinated. Early and accurate prenatal diagnosis, typically via second-trimester ultrasound or fetal echocardiography, is essential for identifying EC and associated defects [16]. It enables timely multidisciplinary planning involving maternal-fetal medicine, neonatology, pediatric cardiology, and surgery. For viable neonates, temporary coverage (e.g., with Bogota bag or pericardial graft) may provide time to assess for staged reconstruction, although long-term outcomes remain guarded.

The diagnosis of Pentalogy of Cantrell can be made during the first trimester of pregnancy utilizing ultrasound imaging (USG). Prenatal diagnosis assists families in making well-informed decisions regarding the pregnancy. In addition, it is also possible to design foetal interventions. Following the delivery, chest X-rays can detect the presence of diaphragmatic hernia and dextrocardia. Postnatal assessment also involves performing CT scans and MRI scans of the heart to evaluate the severity of abnormalities and develop a detailed plan for additional surgical repairs. Echocardiography is crucial for evaluating the cardiac chambers and ejection fraction. Karyotyping is crucial for family counselling because of its connection to aneuploidy [17,18]. In the first case, the diagnosis was confirmed by prenatal ultrasound showing the heart outside the thorax. Intrauterine growth restriction (IUGR) might be caused by various factors such as placental disorders or genetic abnormalities. This condition causing a high-risk pregnancy and delivery, so it requires tight monitoring and adequate medical intervention.

In the second case, the diagnosis based on ultrasound findings shows dextrocardia, ectopic cordis, VSD, pericardial defect, lower sternal cleft, supraumbilical omphalocele, and hypotelorism. These findings are consistent with case report by Fazae et al., indicating that ultrasound revealed a small fetal chest with anterior chest wall defects, indicative of Pentalogy of Cantrell at 23 weeks of gestation. Similarly, Mraihi et al. reported 2 cases: in Case 1, routine ultrasound at 12 weeks of gestation showed the fetal heart outside the chest through a lower sternal defect, along with an omphalocele. In Case 2, ultrasound at 15 weeks of gestation showed defects in the mid-abdominal wall, large omphalocele, diaphragmatic hernia, and ectopic cordis. These findings confirm the accuracy level of the diagnosis for patient case 2, aligning with the clinical pattern documented in the prior case report [19,20]. The congenital defects in the second case can be classified as POC, according to the Toyama classification which meets the criteria for class 2, indicating a probable diagnosis with 4 anomalies present. These anomalies include cardiac abnormalities: dextrocardia, ectopic cordis, VSD, pericardial defect, lower sternal cleft, and an anterior abdominal wall defect in the form of supraumbilical omphalocele. The presence of a diaphragmatic defect has not been ruled out, hence meets the characteristics of partial POC.

The contrasting outcomes in our 2 cases underscore the prognostic significance of several key clinical factors. In Case 1, the neonate had complete ectopic cordis with total displacement of the heart and no thoracic cavity protection, resulting in rapid decompensation from sepsis despite surgical stabilization. The absence of intracardiac defects in this case did not mitigate the severity of the extracorporeal exposure, which left the heart highly vulnerable to trauma and infection. In contrast, Case 2 involved partial ectopic cordis with only partial exteriorization of the heart, and the presence of an intact thoracic cavity allowed for greater hemodynamic stability. Although associated defects such as VSD and omphalocele were present, these anomalies were contained and did not compromise immediate postnatal survival. Furthermore, the timing of intervention—elective delivery at 38 weeks versus emergency delivery at 36 weeks—may have allowed for more comprehensive planning and stabilization in the second case. These findings reinforce that the extent of cardiac exposure, the presence of protective thoracic anatomy, and the timing and coordination of delivery are all critical factors that influence neonatal survival in ectopic cordis.

The recommendation of delivery method for a fetus with partial POC depends on various factors, including the severity of fetus anomalies, associated anomalies, and the expert of the medical team. Generally, a caesarean section (C-section) is preferred to reduce the risk of compression of the exposed heart during vaginal delivery. However, the decision on the mode of delivery should be made by a multidisciplinary team comprising obstetric specialists, pediatric cardiologists, and neonatologists. This team will evaluate the specific circumstances of each case and determines the safest delivery method for both the maternal and the fetus. A case report by Lack et al., reporting a patient with POC thoracoabdominal ectopic and omphalocele, recommend caesarean as delivery method. The reason is due to the potential risks associated with vaginal delivery, including prolonged cardiac compression, damage to herniated organs, and rupture of the atrial diverticulum or omphalocele sac [21].

For future pregnancies planning for both cases with a history of multiple congenital anomalies, a careful and coordinated approach between the medical team is essential. Comprehensive genetic counselling to evaluate the risk of genetic disorders, planning appropriate care with specific medication adjustments, and close monitoring during pregnancy. Consultations with various specialists such as obstetricians, cardiologists, and geneticists are necessary. Comprehensive education is also important, along with emotional and psychological support to planning further pregnancy. The purpose is to minimize the risk of complications and make sure the safety of both the maternal and fetus [21].

This report contributes valuable clinical insight into a rare and life-threatening congenital condition. By presenting both complete and partial forms of ectopic cordis, the study allows direct comparison of phenotypic severity, associated anomalies, and postnatal outcomes. The use of prenatal imaging illustrates the diagnostic pathway and highlights the utility of multidisciplinary planning. The inclusion of a comparative literature table enhances interpretability and contextualizes outcomes relative to previously published cases. The primary limitation is the absence of genetic testing in both cases, which restricts the ability to fully assess the etiology and recurrence risk of EC and associated syndromes. While this was due to resource constraints and parental refusal, it limits understanding of chromosomal or syndromic associations. Genetic evaluation is critical for understanding etiology, recurrence risk, and for guiding future reproductive counseling. We recommend that comprehensive genetic workup be included in future cases whenever feasible. In addition, long-term follow-up data were not available, particularly for the surviving case, which restricts assessment of developmental and surgical outcomes.

## Conclusion

Diagnosis in these cases were made based on the anamnesis, physical examination, antenatal US, and genetic evaluation plan. The second case meets the criteria of POC meanwhile the first case could not be classified. For the next pregnancy plan, complication risk assessment is required with the help of medical team and genetic counsellor.

## Patient consent

We have adhered to ethical guidelines and obtained all necessary permissions and consents.

We obtained written consent from the patient for the publication of this case.

## Appendix Supplementary materials

Supplementary Video 1. Prenatal ultrasound cine loop demonstrating ectopic cordis in a 36-week fetus. The video shows dynamic motion of the fetal heart located completely outside the thoracic cavity. The 4-chamber cardiac structure is visualized with preserved contractility and rhythmicity, unprotected by the pericardium or chest wall. This real-time clip emphasizes the diagnostic value of prenatal sonography in identifying extracorporeal cardiac positioning. Image, video 1
